# Considerations and Guidance for the Structure, Organisation, and Operation of Cardiometabolic Prevention Units: A Consensus Statement of the Inter-American Society of Cardiology

**DOI:** 10.5334/gh.960

**Published:** 2021-04-27

**Authors:** Carlos I. Ponte-Negretti, Fernando Stuardo Wyss, Daniel Piskorz, Álvaro Sosa Liprandi, Alberto Lorenzatti, Livia Machado, Patricio López-Jaramillo, Eduardo Barbosa, José R. Gómez-Mancebo, Ricardo López-Santi, Osiris Valdez, Leonardo Cobos, Adriana Puente-Barragan, Gabriela Borrayo, Emilio Ruiz

**Affiliations:** 1 Unidad Cardiológica del Caribe, Macuto, VE; 2 Unidad de Medicina Cardiometabólica La Floresta, Caracas, VE; 3 Servicios y Tecnología Cardiovascular de Guatemala S.A., CARDIOSOLUTIONS, Guatemala, GT; 4 Instituto de Cardiología Sanatorio Británico SRL de Rosario, Rosario, AR; 5 Department of Cardiovascular, Sanatorio Güemes, Buenos Aires, AR; 6 Instituto Médico DAMIC/Fundación Rusculleda de Investigación y Docencia en Medicina, Córdoba, AR; 7 Unidad Nutrición Clínica, Torre Alfa Santa Sofía, Caracas, VE; 8 Instituto de investigaciones Masira, Facultad de Salud, Universidad de Santander, Bucaramanga, CO; 9 Liga Hipertensão Porto Alegre, Porto Alegre, BR; 10 Hospital Universitario de Caracas and Universidad Central de Venezuela, Caracas, VE; 11 División de Cardiología, Hospital Italiano de La Plata, Buenos Aires, AR; 12 Centro de Especialidades Médicas, La Romana, DO; 13 Hospital El Pino, Santiago, CL; 14 Centro Médico Nacional 20 de Noviembre, ISSSTE, Cuidad de México, MX; 15 Centro Médico Nacional Siglo XXI, Instituto Mexicano del Seguro Social, Ciudad de México, MX; 16 Corporate Medical Affairs Department, Ferrer Internacional, Barcelona, ES

**Keywords:** cardiovascular prevention, cardiometabolic risk, cardiometabolic prevention unit

## Abstract

Cardiovascular diseases (CVDs) remain the leading cause of death worldwide, particularly in low- and middle-income regions such as Latin America. This is because of the combination and interaction in different proportions of a high prevalence of cardiometabolic risk factors and socio-economic and cultural characteristics. This reality brings about the need to change paradigms to consistently and systematically boost cardiovascular prevention as the most cost-effective medium- to long-term strategy to reduce their prevalence in medium- and low-resource countries, not only in Latin America but also in other global regions.

To achieve the therapeutic goals in various diseases, including CVD, the current literature demonstrates that the most effective way is to carry out the patient’s diagnosis and treatment in multidisciplinary units. For this reason, the Inter American Society of Cardiology (IASC) proposes the creation of cardiometabolic prevention units (CMPUs) as a regional initiative exportable throughout the world to standardise cardiovascular prevention based on the best available evidence. This ensures homogeneity in the global management of cardiometabolic risk factors and access to quality medicine independently of the population’s social situation. These guidelines, written by a panel of experts in cardiovascular prevention, defines what a CMPU is, its objectives and the minimum requirements for it, as well as proposing three categories and suggesting an operational scheme. It must be used as a guide for all individuals or centres that, aware of the need for multidisciplinary and standardised work, want to create a unit for the comprehensive management of cardiometabolic risk established as an international research network.

Lastly, the document makes meaningful points on the determination of cardiovascular risk and its importance. These guidelines do not cover specific targets and therapeutic schemes, as these topics will be extensively discussed in another SIAC publication, namely a statement on residual cardiometabolic risk.

## Introduction

Cardiometabolic diseases (CMDs) and their clinical manifestations (i.e., cardiovascular disease [CVD], cerebrovascular disease, peripheral arterial disease, and chronic kidney disease) are, together, the primary cause of morbidity and mortality worldwide [1]. Overall, it is estimated that more than 17 million people die annually from these conditions, representing 31% of all global deaths [1]. CVDs have reached epidemic proportions, and they are associated with very high social and economic costs, both because of substantial direct costs due to healthcare intervention and to significant indirect costs secondary to death and disability [1]. Of note, the CVD’s burden is higher among middle- and low-income countries (MICs and LICs, respectively) than in high-income countries (HICs) [2]. For instance, while CVD accounts for 23% of deaths across HICs, it causes more than 42% of deaths in MICs and LICs. This corresponds to a risk of death from CVD that is between two and four times higher in LICs and MICs than in HICs [2].

The most common pathophysiological substrate in the abovementioned conditions is atherosclerotic cardiovascular disease (ASCVD), which is a pathological process secondary to changes in the arterial wall that encompasses endothelial dysfunction, oxidative stress, inflammation, and atherothrombosis [3]. These changes are the consequence of the sustained and long-term effect of a group of risk factors, together known as cardiometabolic risk factors (CMRFs), which are related to abdominal obesity and ectopic fat and comprise hypertension (HT), dyslipidemia including atherogenic dyslipidemia, diabetes mellitus (DM), and prediabetes. Additional risk factors include modifiable behaviours such as tobacco in all forms (smoking, chewing, vaping), increased alcohol consumption, a sedentary lifestyle, an inadequate diet, and also genetic or hereditary factors [3].

Two standardised case-control international studies on modifiable risk factors associated with myocardial infarction (INTERHEART) and stroke (INTERSTROKE) robustly showed that CMRFs are responsible for more than 80% of CV events [4,5]. Also, both studies demonstrated that these risk factors do not occur in isolation but frequently occur together in individuals and that the sum of distinct factors exponentially increases the risk of either a primary or secondary CV event [4,5]. The notion of cardiometabolic risk (CMR) arises from this scenario, and it is defined as the probability of developing DM, subclinical or clinical ASCVD, or a CV event in the presence of CMRFs.

Current evidence consistently demonstrates that early diagnosis and strict control of CMRFs significantly reduces the probability of a new CV event [6]. In a scenario where most patients have multiple CMRFs, it is reasonable to propose that, for their detection, treatment, and control, the approach should not be conducted in a stand-alone manner, but instead through multidisciplinary risk units where prevention, diagnosis, and management allow a global approach to CMR. These units should ideally consist of a multidisciplinary team of professionals who follow standardised protocols based on the best available scientific evidence, and comprise dedicated clinicians from different specialties, nursing staff, nutritionists, psychologists, and sports therapists, among others. Indeed, implementing a multidisciplinary approach has proven more effective than conventional care as regards the control of risk factors and the reduction of CV risk [7,8,9].

### The Specific Challenges of Latin America

Besides the previous issues, which are common worldwide, Latin America (LATAM) has several sociocultural and historical characteristics worth discussing. This is because these specific challenges contribute to what we define as social risk, namely a higher prevalence of CV events and worse outcomes due to economic and social inequity.

**Figure 1:**
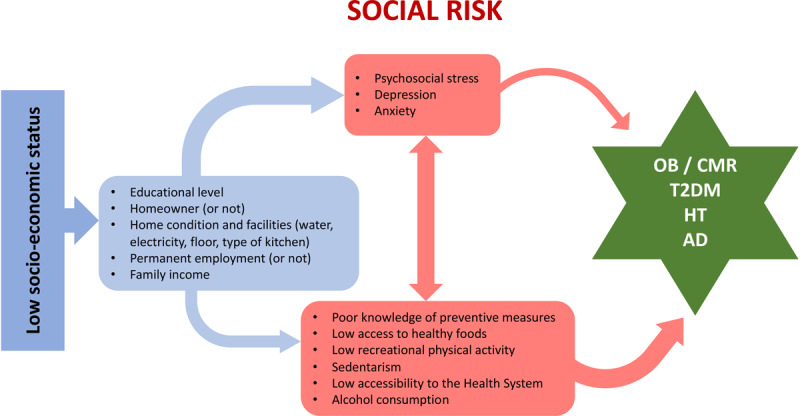
Diagram of the relationship between the socioeconomic status and the development of cardiometabolic conditions. *Footnote:* AD, atherogenic dyslipidemia; CMR, cardiometabolic risk; HT, hypertension; T2DM, type 2 diabetes mellitus.

Twenty-first century Latin America is the result of multiple mixings of Native Americans, Spanish and other European, African, and, more recently, Asian and Middle Eastern ethno-racial groups [10]. This multiethnic and multicultural mixing took place over the past four centuries, and it consists of different proportions depending on each subregion of the continent, such as Mesoamerica, the Andes, the coast, the Southern Pampas or the Amazon, among many others [10]. This mosaic pattern, which can be found in other regions of the world, is also blended with different proportions of educational, cultural, and socioeconomic conditions [11]. In this regard, various observational and cross-sectional studies have consistently shown that there is a relationship between the emergence of CMRFs –and, eventually, with CVD– and demographic, socioeconomic (schooling, educational level, purchasing power), environmental (habitat, nutrition), cultural factors, and inadequate access to quality healthcare (Figure 1) [11,12,13]. Moreover, this socioeconomic reality and its interaction with individuals through history has in turn influenced the expression of the genetic load of human subjects and generates phenotypic manifestations due to epigenetic changes that can be intergenerationally transmitted.

The Whitehall II report is a classic study that assessed the social distribution of central obesity (evaluated using the waist-to-hip ratio) and metabolic syndrome (MS) [14]. The results showed an inverse social gradient, with an increased likelihood of having MS or central obesity among those in the least favourable socioeconomic quintile compared to the those in the top quintile: for central obesity, the increased odds ratio was 2.2 (95% CI: 1.8–2.8) for men and 1.6 (95% CI: 1.1–2.4) for women; for metabolic syndrome, it was 2.2 (95% CI: 1.6–2.9) for men and 2.8 (95% CI: 1.6–4.8) for women.

From the above data, it is clear that, in our setting, it is necessary to guarantee homogeneity in the overall management of CMRFs as well as the access to quality medicine independently of the population’s social stratum. From this setting, we propose that, since CVDs have epidemic behaviour, we must approach them as such. This implies that, if there is an increase in CMD incidence, it merely means that prevention failed. In line with this statement, we must change the paradigm and be more efficient. For this, cardiometabolic prevention (CMP) should be systematically and universally applied and be based on the best available evidence. For instance, randomised clinical trials (RCTs) have provided evidence that therapeutic strategies can reduce the CV risk up to 70% when correctly implemented [15].

In short, CMP entails the accurate application of corrective lifestyle changes and the optimal use of pharmacological treatments to achieve the control of five therapeutic objectives, namely obesity, levels of atherogenic lipoproteins, glucose metabolism, HT, and the prothrombotic state. This approach acknowledges different pathophysiological pathways and identifies therapies that must be considered together, without any hierarchy among them, to achieve an actual reduction in the global risk to provide personalised and precision medicine.

To achieve this ambitious goal, it is necessary to implement a global approach, and this is why the Inter American Society of Cardiology (SIAC) proposes the creation of a network of cardiometabolic prevention units (CMPUs). These are conceived as work units in which a multidisciplinary team works in an integrated and standardised manner based on the best available evidence. The objective is to optimise the diagnosis and degree of control of all CMRFs at once in a comprehensive way, instead of treating them separately and in isolation. Our proposal is for CMPUs to be built on operational and functional concepts, not necessarily implying a single physical space. Their main resource is ‘human,’ and it is paramount that the components of this multidisciplinary team work with well-defined schemes and goals, all under the same standard, have adequate leadership, and also fluent and multilateral communication.

In LATAM, reducing the impact of cardio-metabolic diseases is imperative and why we propose this new way of approaching this epidemic. We believe that the creation of CMPUs may apply to most of the existing health systems of Latin American countries. However, each coordinator will have to make the necessary adjustments to the work system in order to adapt and accommodate each country and community health system. This should be periodically evaluated and make any necessary corrections.

The aim of this Experts’ consensus document written by the IASC is to define how a CMPU should be, as well as its objectives, to propose categories and their minimum requirements, and to recommend an operational scheme that can guide all individuals or centres that, aware of the need for multidisciplinary and standardised work, want to create a unit for the comprehensive management of CMR. Please note that this document does not cover specific targets and therapeutic schemes, since there are many clinical guidelines that deal extensively with the approach to the different CMRFs and each Unit must decide, and periodically update, which guidelines to follow.

## Development of the Consensus Statement

To ensure a best practice approach and a consensus document, the SIAC adopted a modification of the Delphi methodology [16]. This allowed structured and systematic communications, enabling conclusions to be drawn based on evidence- and consensus-based opinions and discussions.

An academic expert panel of 20 members was selected from different Latin American countries, namely Costa Rica, Mexico, Guatemala, Dominican Republic, Venezuela, Ecuador, Peru, Chile, Argentina, Paraguay, and Brazil. The experts were chosen based on their expertise in HT, lipidology, DM, nutrition, and CMD. Besides their proficiency, the group members were required to have no conflict of interest that could influence the discussions’ objectivity on the recommendations.

### Consensus Forming and Content Development

A coordinating committee of 20 experts (including cardiologists, endocrinologists, lipidologists, internists, family physicians, general practitioners, sports therapists, and clinical nutritionists) developed a content index and a list of relevant questions (Online Appendix 1). The panel was organised in balanced working groups that answered the corresponding suggested queries. At a first online discussion meeting, all group members and the corresponding experts prepared a document with potential statements. Afterwards, all the groups met in an in-person meeting to further discuss all questions and answers and prepare the final document. The groups accepted as unanimous those recommendations with a 100% agreement, as consensus, those with at least 80% agreement, and as a discrepancy when there was less than 80% agreement. All the agreed recommendations and conclusions were included in the final report, and a master copy including all the recommendations with arguments for and against was submitted and validated by experts external to the original group in a subsequent last round.

### Background Information and Evidence Review

A non-systematic expert literature search was conducted to search for available literature related to each of the questions and using the following search terms: cardiovascular prevention, cardiometabolic prevention, cardiovascular risk, cardiometabolic risk, dyslipidemia, hypertension, diabetes, prediabetes, obesity, metabolic syndrome, cardiometabolic diseases, risk units, and Latin America. The following bibliographic databases were used: PubMed, SciELO, LILACS, Revencyt, BIREME, ScienTI, LIVECS, and PERIODICA. Non-indexed literature and publications from official or international organisations were only selected if the methodology was considered appropriate. Those publications including epidemiological and clinical research data in nota del revisor the applicability of what is recommended to Latin American countries is less well documented estop creo aplica a responder esta inquietud ademas de otros puntos señalados mas abajo- seguir leyendo- LATAM and/or whose conclusions applied to this region were given priority. Lastly, a drafting committee discarded publications in which a potential bias or conflict of interest was identified. After reviewing a total of 65 manuscripts, 29 were used to prepare the consensus statement.

## Definition of Key Terms

The definition of the following key terms is essential to understanding the CMP process [17]:

- ***Risk factor:*** *a* condition that precedes the onset of a disease or its outcomes, it is statistically correlated with them, shows predictive power, and it is a plausible pathogenetic mechanism.
- ***Goal:*** it is the ideal level (quantitative or qualitative) that any modifiable or controllable risk factor should reach so that, when applying prevention measures, the risk of suffering a CV event is minimised.
- ***Prevention:*** long-term intervention on one or several risk factors to prevent or delay the development of a disease or, if already present, to avoid sequelae or death.
- ***Primordial prevention:*** behaviour aimed at preventing the appearance of any modifiable risk factor in subjects without evidence of established clinical disease through the population, family, and individual education strategies. These population-based measures of health prevention and promotion are mainly aimed at stimulating the adoption of healthy lifestyle behaviours to avoid the occurrence of risk factors for developing a particular disease.
- ***Primary prevention:*** long-term intervention in individuals at risk who have not yet developed the disease at the clinical level. It is also defined as any intervention or behavioural modification that tends to reduce, control, or stop risk factors for developing the disease to prevent or delay its appearance.
- ***Secondary prevention:*** intervention in patients with an established clinical condition to avoid the recurrence of the episode or the appearance of another related event, and of preventing the progression of the disease, disability, or death of the individual.
- ***Cut-off point for intervention:*** limit value or threshold of a risk factor above which it is necessary to initiate actions to achieve optimal therapeutic goals.
- ***Overall CV risk:*** a valid estimate of the probability of developing a CV event over five, ten, or more years in individuals without clinical manifestations of CV disease at the time of the examination.

## Definition and Requirements of a CMPU

### Definition and notion of CMPUs

A CMPU is an operational and functional concept whose main resource is human, so it does not necessarily imply working in a single physical space. The key factor is that those included in the multidisciplinary team work in an integrated manner, all under the same standard, with well-defined objectives, proper leadership, and fluent and multidirectional communication. Hence, it follows that, for instance, depending on the increasing complexity of the different Units (see below), optimal prevention and work can be done from either a small consulting room -with one physician and one nurse or trained and certified medical assistant- or from a more complex CMPU that is integrated into a medical centre, with the possibility of having all human and technological resources at its disposal. Still, it is important that the CMPU is integrated and connected to the health network to allow for the patient’s referral and cross-referral depending on each particular case’s complexity and needs. An additional advantage of belonging to the health network is the current and growing possibility to interact online with other professionals to resolve doubts about the patient’s management straightaway.

One of the challenges of the CMPU team is to integrate their work scheme into each country and community health system. Therefore, it is essential to harmonize with the authorities and the local health system prior to initiating the development of each unit. This will allow working in a coordinated and articulated manner integrated into the existing working procedures without generating frictions and interferences with the other medical community members. These are necessary steps to implement a confident reference system and a counter-referral that will benefit the patient and be more efficient and cost-effective at the end of the day.

Besides medical care, and as an essential part of the CMPU activities, they must develop adequate registry systems with two primary purposes. The first one is to know and self-assess its results and effectiveness. This must include the objective evaluation of the cost effectivity of the integrated UPCM work within the health system and the capability to analise whether the UPCM work provides added value to the current health system. For this, CMPUs must have a common methodology, designed and validated to periodically evaluate whether the goals were achieved in each patient and the population, including the rate of compliance with the therapeutic measures -both pharmacological and non-pharmacological. This way, it is possible to monitor the work done and make timely and necessary corrections of the least effective interventions, analyse how the new work scheme of integrating UPCM and health system could be more efficient in terms of objectives’ achievement and results and, conversely, reinforcing the accurate ones to optimise their work and improve the accomplishment of results. The second essential objective is to generate data that can be used effectively in epidemiological or clinical research. In parallel with this, CMPUs should ideally have, as part of their objectives and to complement their healthcare activity, teaching activities for other health professionals in training, patients, and the community.

An effective CMPU must be an overall objective and go beyond the medical offices or healthcare institutions’ physical space. CMPUs must get involved with the community and seek solutions; the modern notion of health must evolve and expand the scope and, as a consequence, propose and be part of the solution to specific problems. Consistent with this consideration, CMPUs must organise activities that involve the community, which can be educational, investigational, or risk factor screening, among others. These activities must include children and young populations, as it has been recently demonstrated that effective educational interventions and healthy behaviours in children and adolescents have to two major effects: firstly, those healthy habits acquired at an early age are integrated into daily behaviour more reliably and easily, and thus are more effective and durable. Secondly, children can be vectors of change towards their teachers, homes, and the community [18].

As detailed in the following sections, three types of CMPU are proposed, mainly depending on their capacity to manage patients with different complexities and available infrastructure (Table 1). Although overall it is not feasible to define the patients that each type of CMPU can take care of, it is possible to give general rules for the management in each of the types. Generically, as the CMPU increases its capacity (in terms of staff and infrastructure), the patient’s complexity that can be treated increases as well, with CMPU type 3 being the one able to manage the most complex patients.

**Table 1:** Characteristics and requirements of the different types of cardiometabolic prevention units according to degree of complexity.

Unit Type	Objective	Coordinating Medical Staff (any of these)	Non-medical staff	Basic equipment	Optional equipment

Type 1	Primordial prevention Primary prevention of **low and intermediate risk**	General practitioner Family physician Internist Cardiologist Endocrinologist	Nursing staff or medical assistant	Tensiometer Balance Measuring tape	Electrocardiograph* Glucometer Dynamometer
Type 2	Primary prevention of **intermediate and high risk**	Internist Cardiologist Endocrinologist	Nurse or medical assistant* Clinical nutritionist Psychologist*	Sphygmomanometer Balance Measuring tape Electrocardiograph Echocardiograph * ABPM*	Glucometer Dynamometer Arterial 3D ultrasound* Possibility of ABI measurement*
Type 3	Secondary prevention AND Primary prevention of **intermediate, high and very high risk**	Cardiologist	Nurse or medical assistant Clinical nutritionist* Psychologist* Physical trainer or sports therapist	Sphygmomanometer Balance Measuring tape Electrocardiograph Echocardiograph * ABPM* Possibility of ABI measurement	Glucometer Dynamometer Ultra 3D arterial sound* CT for coronary calcium measurement*

* On site or available for reference at the centre and that works in an integrated way with the protocol of the Unit. ABPM, ambulatory blood pressure monitoring; CT, computed axial tomography; 3D, 3-dimensional; ABI, ankle-brachial index arm.

### Requirements of a CMPU

Given the diversity of health systems and each country’s reality from the sociodemographic perspective, this expert panel defined the minimum requirements for each type of Unit (Table 1). Subsequently, each Unit must adapt these requirements to its real world.

#### Human Resources: Medical and Non-Medical Staff

The CMPUs must consist of an interdisciplinary group of human talent that includes a physician as the group leader, preferably a specialist in cardiology, internal medicine, or endocrinology. In the absence of a specialist, the team may be led by a general practitioner or a family physician provided that he/she is accredited through certified training or has at least three years of practical experience in managing CV promotion and prevention programmes.

Depending on the type, the CMPU will need professional nursing staff or an experienced medical assistant with adequate expertise in the management of CMRFs, as well as a clinical nutritionist, a psychologist, and a physical trainer or sports therapist. Also, the team must have, depending on its original conformation, external consultants specialised in endocrinology, cardiology, nephrology, and neurology. These personnel must be integrated into the patient’s standardised approach and have knowledge on treatment goals and objectives. Finally, it is an absolute requirement for all types of units that all personnel involved in the patient’s care is adequately trained and skilled in CMP. Verifying this is the responsibility of the Unit Coordinator.

When possible, and depending on each Unit’s sociodemographic characteristics, we suggest giving preference to personnel from the community where the CMPU is established. This will allow better interaction between the Unit and the population of its area of influence.

#### Physical Infrastructure/Equipment

The facilities of the CMPU, no matter if it is part of a Hospital Unit, a Health Centre, or an independent Unit, should ideally have clinical offices for the physician, the clinical nutritionist, and the psychologist, as well as a central nurse station. When this is not possible, the CMPU must at least have two clinical offices, one for the leading physician and the other to be used by the nursing staff or medical assistant.

All offices should ideally have access to electronic medical records and, nowadays, and increasingly in the future, enough technology to allow telemedicine consultations. Likewise, CMPUs must have access to a clinical laboratory and imaging service. It is important to clarify that these diagnostic support services (clinical laboratory and imaging) should always be the same and must have unique and defined protocols agreed between the CMPU and the respective diagnostic support service. Moreover, they must be adequately standardised and periodically certified according to the regulations of each region or country.

Lastly, CMP involves behavioural changes, and it is essential to achieve success in this process, the education of the patients, their relatives, and caregivers. This is why the office must be tailored to accommodate posters, written messages, blackboards, billboards, brochures, and even videos through monitor displays.

## Specifications and Structure of the Different Types of CMPUs

### CMPU Type 1

**Objective:** This type of Unit’s fundamental purpose is CV prevention in people at low/moderate risk. However, they can manage other types of patients as long as the characteristics of the health personnel and the infrastructure techniques allow that, and other centres of the area of influence cannot manage complex patients.

The patient can be admitted to CMPU type 1 from:

- Triage (*de novo* patients), that is, diagnosed in the consultation office itself
- CMRF screening programmes conducted in the community
- Referred by a primary care physician not committed to CV prevention

**Minimum required staff:** One physician and nursing staff member or medical assistant.

#### Diagnostic requirements

***Basic diagnostic equipment:*** A validated and calibrated sphygmomanometer (preferably electronic), a scale, and a measuring tape. Lastly, it is essential to have access to a clinical analysis laboratory in the same area or close to the Unit.

***Optional diagnostic equipment:*** Electrocardiograph, glucometer, and dynamometer.

### CMPU type 2

**Objective:** To control overall risk factors in patients at low, moderate, and high risk. We define high risk as two or more risk factors, a difficult to manage risk factor, a patient with target organ injury, or stable after a CV event.

The patient can be admitted to CMPU type 2 from:

- A triage of patients with suspected CV risk (*de novo* patients) in the absence of a close CMPU type 1.
- A coronary or stroke care unit, or after hospitalisation because of an acute CV or cerebrovascular event.
- Other hospital or clinic services, or nearby Centres or Units, when the diagnosis or specialised control of one or more CV risk factors require a comprehensive and integrated approach.

#### Personnel

**Minimum required staff:** In this case, the human resources need to be more complex; the coordinating physician must be a specialist in cardiology, internal medicine, or endocrinology. Other non-specialist physicians may also work in the team.

**Non-medical staff:** In addition to a nurse or medical assistant, these CMPUs must have, or have access to, a clinical nutritionist and a psychologist, either located in the Unit or available for reference at the centre where it is located. Besides, it is essential that the Unit always works with the same professional, who must be involved in the Unit’s work methodology, goals, and objectives.

**Optional additional staff:** A physical trainer or sports therapist. As with the psychologist, he/she must always be the same professional and needs to be available in the same Unit or the centre where it is located and must be in line with the Unit’s work procedures.

#### Diagnostic requirements

***Basic diagnostic equipment:*** Besides the sphygmomanometer, the scale, and the measuring tape, the Unit must have an electrocardiograph, and the possibility of performing an echocardiogram and 24-hour blood pressure monitoring (ABPM), in addition to the measurement of the handgrip strength in patients who warrant it. Lastly, it is essential to have access to a clinical analysis laboratory in the same area or close to the Unit.

***Optional diagnostic equipment:*** Glucometer, dynamometer, and accessibility or possibility to conduct a 3D carotid and/or femoral ultrasound.

### CMPU type 3

**Objective:** Type 3 units are more complex and function as specialised reference centres. The priority population are patients with high or very high CV risk that are difficult to manage or who recently had a CV event, including postoperative patients who underwent coronary revascularisation surgery. However, the Unit could admit patients at lower risk if there is no nearby CMPU or they are referred for control and follow-up.

**Minimum required staff:** In these units, the human resource needs to be the most complex one. The coordinating physician must be a certified cardiologist, although other medical specialists can also be part of the team. Moreover, and in addition to nursing personnel, the Unit must include a clinical nutritionist, a psychologist, and a physical trainer or sports therapist.

#### Diagnostic requirements

***Basic diagnostic equipment:*** Besides the sphygmomanometer, scale, and measuring tape, the Unit must have an electrocardiograph and the possibility of performing an echocardiogram and ABPM, in addition to the measurement of handgrip strength for patients who require it. It is also essential to have access, within or close to the Centre, to a clinical analysis laboratory.

***Optional diagnostic equipment:*** Glucometer, dynamometer, 3D carotid and/or femoral ultrasound, and computerised axial tomography (CAT) to measure coronary calcium. Any other available diagnostic technology (e.g., imaging, serological, immunological, genetic, etc.) will always be helpful. What is most important about these more advanced techniques is that appropriate ethics always need to be implemented when they are used, considering the cost-effectiveness ratio and they must be detailed and explained to the patient with his/her individual informed consent, and never employed for experimental purposes.

## Operational Recommendations and Work Scheme of the CMPUS

**Figure 2:**
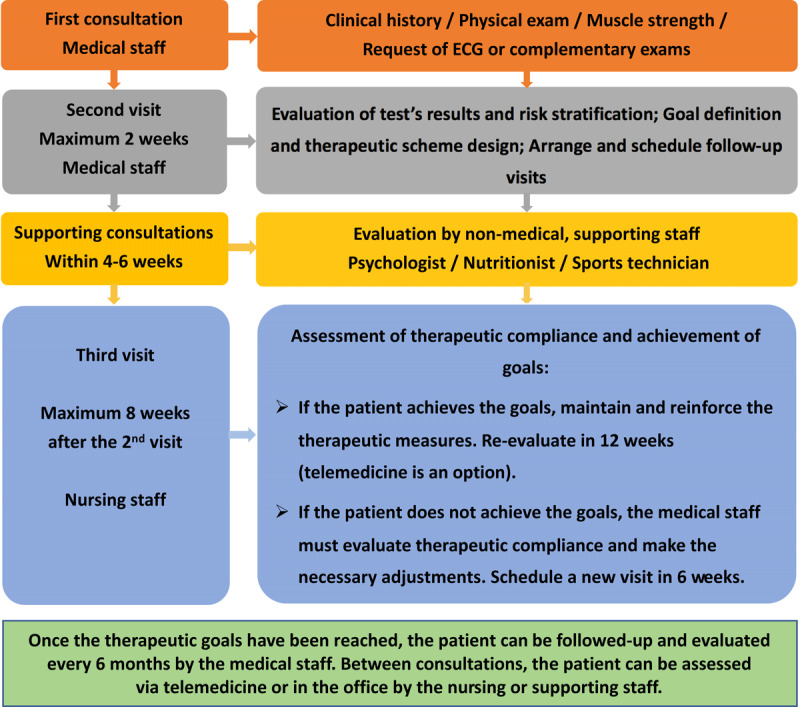
Diagram of the fundamental steps and periodical evaluations to be performed by cardiometabolic units. *Footnote:* AD, atherogenic dyslipidemia; CMR, cardiometabolic risk; HT, hypertension; OB, obesity; T2DM type 2 Diabetes Mellitus.

Cardiometabolic prevention is based on three fundamental steps (Figure 2): 1) early detection of CMRF and calculation of each patient’s global risk; 2) once the risk is defined, to design and apply the correct therapeutic intervention based on the best available evidence, defining specific targets and therapies; and 3) to periodically evaluate the effectiveness of the prescribed therapy on individual risk factors, verifying that the patient has reached the pre-established goals and reinforcing compliance with the treatment. When targets are achieved, act accordingly, highlighting the need to maintain compliance or making the necessary changes if the predefined goals are not reached. At this point, it must be stressed that adherence to the therapeutic scheme must be systematically investigated. This is particularly important in patients on multiple drugs, which is indeed the majority of patients in a CMPU, because the lack of therapeutic adherence can increase the risk of a new atherosclerotic CV event by up to 36% [19]. Certainly, non-adherence should be considered as a risk factor for a new event and, when detected, strategies must be implemented to reduce it, including the following:

1. Simplify the therapeutic scheme by reducing the number of tablets per day using polypills that include the drugs already prescribed in the original patient’s therapeutic plan, as CV polypills have demonstrated improved risk factor control [20].
2. Implement education initiatives to increase disease awareness, awareness of the risk involved, and how treatment can decrease it.
3. Guarantee accessibility and availability of the prescribed drugs.
4. Incorporate the use of new technologies such as telemedicine, text messaging, mobile applications, etc.

We have to make it absolutely clear that the ultimate and fundamental objective of the whole process is the maximum possible reduction of the global risk for new CV events, always based on the best available evidence. Therefore, the concrete steps to meet this objective encompass a unique and well-defined operational structure where the therapeutic goals and schemes are in line with the chosen local or regional guidelines. Moreover, the actions to be carried out in each consultation, the time between each visit, which medical examinations to request before each consultation, and when to refer to other specialties or centres, must also be clearly defined.

### Actions to be carried out in each consultation

It is important to bring together activities in the same single visit and, if possible, organise the appointments for other consultations (nutritionist and psychologist, for example) to take place on the same day, as this will help the patient meet the predefined goals. It is also important to note that it is not essential for a physician to visit the patient in all consultations. For instance, visits for test reviews, evaluation of results, weighing, etc. can be completed by an appropriately trained and supervised nurse or medical assistant, as they will be qualified to evaluate results and take the appropriate actions with the support of the medical personnel in the event of any doubt or eventuality. Also, in order to simplify work and reduce overcrowding of medical offices, it is suggested to implement a telemedicine system for follow-up controls and therapeutic orientation.

## Risk Stratification

This section includes some details regarding risk stratification and describes the minimum tests and complementary examinations that might be available at the CMPUs.

Cardiovascular risk stratification, using one of the risk scores that best suits the most prevalent risk factors in the LATAM population, is key in evaluating all patients to choose the best approach based on the estimated risk. Additionally, it has the added value of engaging the patients in the therapeutic process by knowing their individual risk.

It is important to always apply the same methodology to stratify risk, as this allows us to adequately assess the CV risk change in each patient and the population. However, the application of any risk score has the limitation that it is a snapshot of the individual risk and does not consider the risk’s dynamic conditions over time.

### Key points to keep in mind when stratifying risk

- CV risk is a dynamic process, so it should be periodically assessed when the patient’s clinical conditions change or, ideally, in each follow-up visit.
- New risk factors, such as psychosocial stress and socioeconomic disparity in access to healthcare systems, must be specially considered when managing patients in LATAM and other regions around the world with similar economic characteristics.
- In intermediate-risk patients, who represent a significant group of the population, it is necessary to use other complementary methods to reclassify them into high- or low-risk.
- Since CVD is a process that begins early in life, we believe that, regardless of age, all patient risk must be stratified.

### Minimum complementary exams

The completion of complementary exams is essential to assess the CV risk profile. The minimum complementary exams that every CMPU must be able to complete are described below.

#### Laboratory tests

##### Blood and urine biochemical parameters

- To assess the lipid profile, include the measurement of total cholesterol (TC), triglycerides (TG), high-density lipoprotein cholesterol (HDL-c), and low-density lipoprotein cholesterol (LDL-c). For LDL-c assessment, we suggest using the Martins/Hopkins estimation [21], since this formula is more accurate when TG are higher than 150 mg/dL, which is frequent in patients from LATAM, where atherogenic dyslipidemia is prevalent. Lastly, non-HDL must be calculated as well.
- To assess glucose metabolism, fasting plasma glycemia must initially be measured. In cases where insulin resistance is suspected, an oral glucose tolerance test (two hours after the intake of 75 g of glucose) must be conducted.
- The routine use of glycated haemoglobin (HbA1c) for the diagnosis of type 2 DM or prediabetes is not recommended.
- To assess renal function, measure plasma creatinine, and always calculate the estimated glomerular filtration rate. It is important that, in consecutive visits, the same formula is used.

***Haematology parameters:*** Cell blood count, including white blood cells (WBC), red blood cells (RBC), and platelet count. Also, include WBC differential count, haemoglobin concentration, and haematocrit.

#### Imaging techniques

In patients with intermediate CV risk, imaging studies can help reclassify risk or provide information to make the best decision regarding goals and treatment. Besides, the fact that the patient objectively perceives the disease helps to improve therapy compliance.

#### Coronary Tomography

The coronary arterial calcium (CAC) score, measured through computed tomography (CT), has proven useful in identifying patients and guiding treatment, and this includes the Hispanic population [22,23]. It is not strictly correct, from the evidence-based medicine perspective, to extrapolate the benefit of CAC to patients in LATAM and the Caribbean. However, we can likewise say that patients with CAC ≥100 Agatston units (AU) are at higher risk than those with CAC=0 AU. Thus, we recommend performing CAC scoring in those centres where there is availability, as long as the cost-benefit ratio justifies it and the patient or family members agree.

#### Carotid Doppler Ultrasound

Carotid intima-media thickness (c-IMT) does not appear to be a good predictor of CV events, and therefore this guide does not recommend its use to assess the patient’s CV risk systematically. By contrast, the presence of atherosclerotic plaques in the femoral/carotid territories evaluated by 3D ultrasound is a validated methodology for diagnosing subclinical ASCVD and a risk predictor [24,25]. It should be emphasised that the plaques are detected earlier in the femoral artery than in the carotid territory [24,25].

The ideal situation would be to conduct both the CAC and the 3D arterial ultrasound techniques since concomitant use increases the capacity to diagnose subclinical atherosclerosis and predict new CV events. However, each study’s associated costs and complexity favour 3D ultrasound [26], and this is why we recommend it as the method of choice to detect and diagnose subclinical ASCVD. That being said, none of these methods is indicated for all patients, but only for those at intermediate risk and conducted in centres with certified expertise, when the cost-benefit ratio justifies it, and if the patient agrees. Lastly, these techniques should never be used experimentally without adequate consent from the patient or their family members.

#### Other methods

##### Artery stiffness

This parameter, which expresses the arteries’ aging and the loss of elastic fibres and their associated compliance, is measured by the pulse wave velocity or the augmentation index (AI). An increase in arterial stiffness is related to damage to the vascular wall. A value of 12 m/s is considered the cut-off point associated with alterations in aortic function in hypertensive patients [27]. Arterial stiffness improves the possibility of CV risk stratification, but its systematic use is not recommended.

##### Ankle-brachial index

The ankle-brachial index (ABI) is a simple and reproducible test that allows asymptomatic atherosclerotic disease to be detected. An index of <0.9 indicates stenosis of more than 50% between the aorta and the leg’s distal arteries with a sensitivity of 79% and a specificity of 90% [28]. Moreover, it allows patients with peripheral arterial disease who are even asymptomatic to be identified. The index is inversely related to CV risk, but there is still controversy about whether it enables patients to be reclassified [29]. Given the ease of performing the ABI, it is recommended that in all CMPUs there is the possibility of doing it for the detection of asymptomatic atherosclerotic disease.

## Final Recommendations

The purpose of this publication is to provide guidance for all those interested in creating a CMPU. However, each one must adapt these recommendations to their local situation while following the guidelines described here.

We also wish to stimulate the creation of a CMPU network whose operating scheme is standard to exchange successful experiences and report execution failures to give each other mutual feedback on this interaction. We also hope that the CMPUs provide important epidemiological information so we can better understand the impact of CMRF on regional cardiovascular morbidity and mortality, as well as evidence on how to reduce its effects.

## Additional File

The additional file for this article can be found as follows:

10.5334/gh.960.s1 Online Appendix 1. Key Questions answered and points developed by the group of experts.
