# Supplementary material for: Considerations and Guidance for the Structure, Organisation, and Operation of Cardiometabolic Prevention Units: A Consensus Statement of the Inter-American Society of Cardiology
Source: Glob Heart. 2021 Apr 27;16(1):27. doi: 10.5334/gh.960 (PMC8086731; doi:10.5334/gh.960)
Supplement: Online Appendix 1. — Key Questions answered and points developed by the group of experts. [file gh-16-1-960-s1.pdf]

Online Appendix 1: Key Questions answered and points developed by the group of experts.

1. Rationale and objectives of the global cardiovascular risk prevention units

1.1. Why is it necessary to create multidisciplinary cardiometabolic prevention units (CMPUs)

1.2. CMPU's cost-benefit ratio

1.3. CMPU's objectives

1.4. Patients who must be managed in the CMPU. Primary or secondary prevention?

1.5. Priorities of care in the CMPUs

1.6. Organisational chart of work, minimum necessary staff (e.g., Medical Doctor, nurses, nutritionists, psychologists, educators, sports technicians?) and suggestion of physical space needed.

1.7. Flowchart that patients must follow from admission to follow-up, such as how often should they be scheduled for a visit, what should they do at each visit, and for how long should they visit?

2. Risk stratification

2.1. In which patients is it necessary to determine risk?

2.2. Importance of risk stratification as an initial step in the management of cardiovascular (CV) prevention. Is it necessary? Why?

2.3. What is the best method to stratify CV risk in Latin America (i.e., by risk tables, images, subclinical atherosclerosis assessment, or by symptoms and risk factors?)

2.4. Is lifetime CV risk justified?

2.5. Steps to detect CV risk

2.6. Medical history, minimal and advanced or second-line complementary exams

3. Arterial hypertension.

3.1. Simplified care guidelines for patients with hypertension

4. Dyslipidemia

4.1. Atherogenic dyslipidemia as a cause of residual CV risk

4.2. Role of combinations and new agents

5. Prediabetes and Diabetes Mellitus

5.1. Diagnosis of prediabetes and diabetes mellitus (DM)

5.2. Need, standardisation, and role of the oral glucose tolerance test (OGTT) in coronary patients

5.3. Standardisation of glycosylated haemoglobin

6. Lack of adherence as a risk factor. How to combat it?

6.1. Lack of adherence as a risk factor component of global risk

6.2. Strategies to combat lack of adherence

6.3. The polypill as a strategy to reduce lack of adherence and reduce global risk
